# Variation of Hydrothermal Conditions under Climate Change in Naqu Prefecture, Tibet Plateau, China

**DOI:** 10.3390/ijerph15102271

**Published:** 2018-10-16

**Authors:** Boya Gong, Baisha Weng, Denghua Yan, Tianling Qin, Hao Wang, Wuxia Bi

**Affiliations:** 1 State Key Laboratory of Simulation and Regulation of Water Cycle in River Basin, China Institute of Water Resources and Hydropower Research, 1-A Fuxing Road, Haidian District, Beijing 100038, China; gongboya@126.com (B.G.); baishaweng@126.com (B.W.); qintl@iwhr.com (T.Q.); wanghao@iwhr.com (H.W.); biwuxia_1992@163.com (W.B.); 2 Department of Water Resources, China Institute of Water Resources and Hydropower Research, A-922, 1 Yuyuantan South Road, Haidian District, Beijing 100038, China; 3 College of Hydrology and Water Resources, Hohai University, Nanjing 210098, China

**Keywords:** hydrothermal conditions, precipitation, accumulated temperature, vegetation growth, Naqu Prefecture, Tibet Plateau

## Abstract

Analysis of the suitability of hydrothermal conditions for vegetation growth would benefit the ecological barrier construction, water resources protection and climate change adaptation. The suitability of hydrothermal conditions in Naqu Prefecture was studied based on the spatial displacement of 500 mm precipitation and 2000 °C accumulated temperature contours. Results showed that the 500 mm precipitation contour had a shifting trend toward the southwest, with a 3.3-year and 7.1-year period, respectively, in the longitudinal and latitudinal direction, and the longitude changed suddenly around 1996. The 2000 °C accumulated temperature contour had a shifting trend toward the northwest, with a 1.8-year period and a 7-year sub-period in the longitudinal direction; the longitude had a catastrophe point between 1966 and 1967, while the latitude had a catastrophe point between 2005 and 2006. When located in the same vegetation zone, the annual precipitation in Naqu Prefecture was higher than the national average, while the accumulated temperature was lower than the national average, indicating that areas with suitable hydrothermal conditions suitable for vegetation growth showed a northwestward shift tendency. This research would help to support some recommendations for plants’ ecological system protection in alpine areas, and also provide guidelines for climate change adaptation.

## 1. Introduction

Naqu Prefecture is located at the intersection of the South China Sea and the Indian Ocean water vapor streams. It is significantly affected by climate change, thus profoundly affecting the dynamics of the surface circulation field and the changes in surface cover. Naqu Prefecture is the water resource conservation areas and ecological barrier construction area of the Nujiang River and Eastern Tibet. However, it has a strong dependence on hydrothermal conditions with fragile vegetation. It is of great importance to analyze the suitability of hydrothermal conditions for vegetation growth in this area. This study can provide references for the ecological barrier construction, water resources protection and addressing the effects of climate change.

Temperature and precipitation are important criteria for measuring regional hydrothermal conditions [1,2]. Changes in temperature and precipitation can produce important geographic, environmental, resource, agronomic, economic and sociological effects [3,4,5,6,7,8,9]. Accumulated temperature can reflect the temperature impact on vegetation growth from two aspects, action time and action intensity, hence, the change of accumulated temperature is more essential to vegetation change. Previous studies have shown that 10 °C is the lower limit temperature for the growth of most vegetation, and an accumulated temperature of more than 10 °C is an important factor affecting vegetation growth and a key caloric indicator for measuring crop yields [10,11].

Most of the previous studies on precipitation focused on the change of annual precipitation and the 400 mm precipitation contour [12]. The main reason is that the 400 mm precipitation contour is the demarcation line between semi-humid and semi-arid regions, forest vegetation and grassland, cultivated civilization and nomadic civilization, planting and animal husbandry in China [13,14,15,16]. Located in the alpine region of the Tibet Plateau, Naqu Prefecture has unique climate and vegetation characteristics. Moreover, there was only few coniferous forests distributed in areas with annual precipitation of more than 500 mm in the region. According to related research [17] on the accumulated temperature demand of vegetation in alpine areas, 2000 °C accumulated temperature (of those daily temperature higher than 10 °C) was needed yearly during the whole growth season of vegetation. Hence, we choose the 500 mm precipitation and 2000 °C accumulated temperature as the threshold of the suitable hydrothermal conditions in this study.

The opinions for planting trees in Naqu Prefecture vary at present in China, and academicians have had a heated discussion about this too [18]. Some researchers, such as academician Sun Honglie, hold the opinion that it was impossible to plant trees in Naqu Prefecture, saying that “I don’t dare to agree …”, while academician Zheng Du holds that “a human plan that is not based on [the] great laws of Nature will only bring disaster”. Some researchers hold the opposite view, like Professor Wang Yanfen who has said that “it is almost impossible to plant trees in large areas, but it is possible to try to plant small shrubs in local areas …”. Some experiments have been carried out, and there are also some successful cases [19], albeit affected by too many natural and artificial factors, so the feasibility of planting should be analyzed from the aspects of hydrothermal, solar, and soil conditions, etc.

The main objectives of our study were to: (i) reveal the changes of annual precipitation and accumulated temperature (of those daily temperature higher than 10 °C); (ii) analyze the spatial characteristics of the 500 mm precipitation and the 2000 °C accumulated temperature; (iii) describe the response of the ecosystem to them from the aspects of trend, periodicity and abruptness systematically. Our study provides support for related research on the spatial change of vegetation types, and also provides references for the ecological environment management in the Tibetan Plateau, where is a lack of data and information.

## 2. Materials and Methods

### 2.1. Study Site

**Figure 1:**
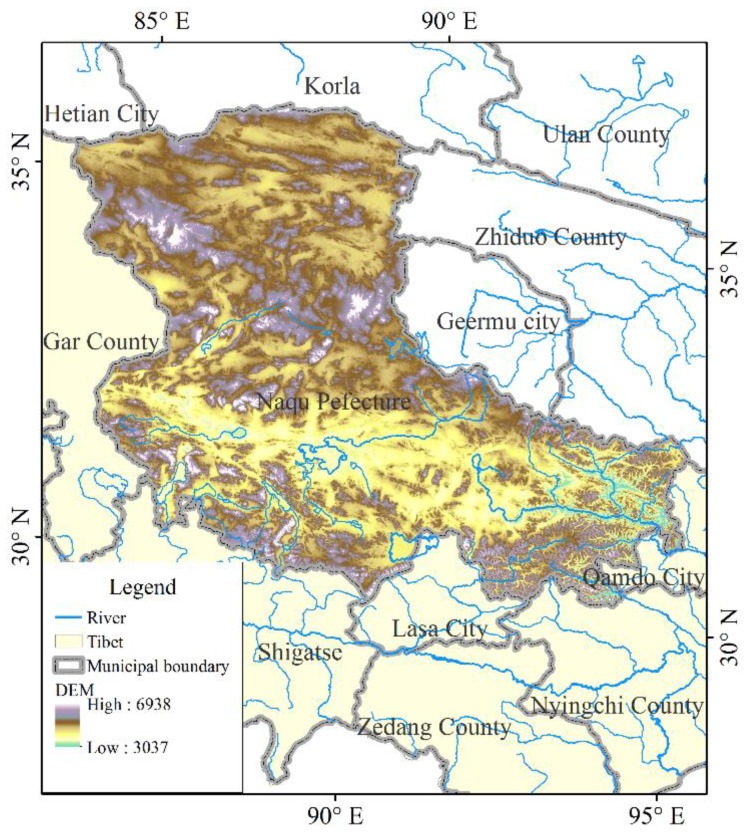
The Naqu Prefecture.

Naqu Prefecture is located at the northern part of the Tibet Autonomous Region, the Central Tibetan Plateau, among the Tanggula Mountains in the north of Tibet, the Nyenchenthanglha Mountains and the Kailas Range. It is the headwater region of many large rivers such as the Yangtze River, the Nu River, the Lhasa River and the Yigong River (Figure 1). Naqu Prefecture has an average elevation of over 4500 m. The northern part is a typical plateau mountainous terrain, while the eastern part is plateau mountainous area, and the southern part is located at the confluence zone of the northern Tibetan Plateau and the eastern Tibetan alpine valley. From 1956 to 2011, the average annual precipitation was 330 mm, the accumulated temperature was 1240 °C, so the heat is insufficient, the climate is cold and dry, and the main vegetation types are alpine grassland and alpine meadow. The vegetation zones distributed in Naqu Prefecture include the alpine grassland zone, alpine shrub meadow zone, alpine meadow zone, subtropical mountainous cold-temperate coniferous forest zone, alpine desert zone, and warm grassland zone. The data was downloaded from the Vegetation Regionalization Map of China provided by the Resource and Environment Data Cloud platform of Chinese Academy of Sciences (http://www.resdc.cn) [19].

### 2.2. Meteorological Data

**Figure 2:**
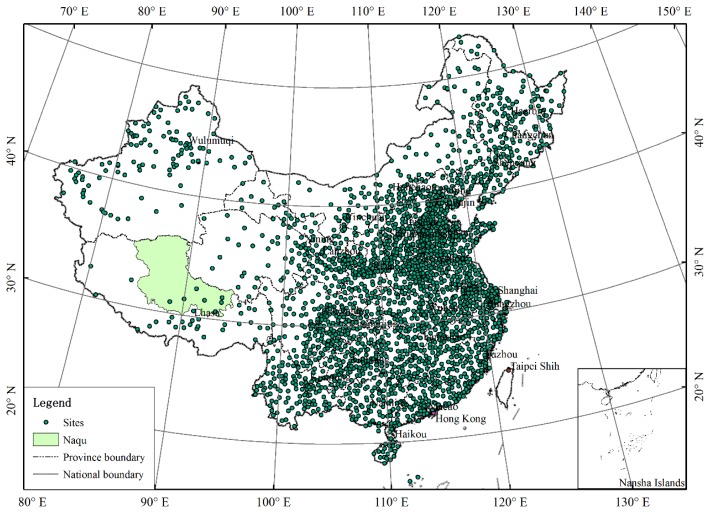
The location map of 2470 meteorological stations in China.

The most commonly used temperature and precipitation data at present is the TRMM data and observed meteorological data. For the TRMM 3B43 data, there was an obvious deviation for the Tibetan Plateau [20], so a Kriging interpolation method has been sometimes chosen to improve the accuracy of TRMM data [21]. At the same time, the spatial resolution of TRMM data is relatively low, so we must downscale it to do our research and to avoid resampling errors and we use the interpolation data of observed meteorological data directly in this work to further explore the time-space distribution of precipitation and temperature. Daily precipitation and temperature were collected from 1956 to 2011 from 2470 meteorological stations located nationwide in China and provided by the China Meteorological Administration (http://cdc.cma.gov.cn/). As the data series from 1961 to 2011 are relatively complete, the observed temperature and precipitation in this period were selected as the basic analytic data in this study. For missing data, limited meteorological station data in adjacent spatial domains in the basin to achieve scale extension of time dimension of temperature and precipitation was utilized [22]. The central difference method (CDM) was used for the interpolation of single missing data values, while multiple consecutive missing data were interpolated by computing the perennial average value of those days [23]. After extending the missing data, the basic data was further reorganized to obtain the accumulated temperature and annual precipitation of each station. Figure 2 shows the distribution of meteorological stations nationwide in China. This study selected Naqu Prefecture as the study site, while the density of meteorological stations in southwestern China is relatively small. Therefore, we firstly obtained the nationwide raster data by interpolating the data of national meteorological stations, and then we got the annual precipitation and accumulated temperature raster data of Naqu Prefecture by resampling the nationwide data, so that we can do our later study of Naqu Prefecture.

### 2.3. Contour Generation and Quantification of Spatial Position

By comparing various spatial interpolation methods, we finally chose the Co-Kriging interpolation method (COK) which has been widely used in practice [24,25,26]. We interpolated the raster surface with a spatial resolution of 5 km × 5 km from points data of the annual accumulated temperature and precipitation by using the Kriging tool of ArcGIS (Version 10.2, Environmental Systems Research Institute, Redlands, CA, USA). Based on the raster surface of the accumulated temperature and precipitation, the Contour tool in the ArcGIS toolbox was used to generate the 2000 °C accumulated temperature and the 500 mm precipitation contours, then the most continuous contours with the largest distribution of the latitude and longitude were finally selected.

In this study, the weighted average space coordinates of the precipitation contours were used to characterize their spatial positions. On the ArcGIS platform, the accumulated temperature and precipitation contours were discretized into points, and the weighted average coordinates of the latitude and longitude of these discrete points were calculated, which were the weighted average space coordinates we used. The annual change of the weighted average position (center point coordinates) of the contour lines reflects the holistic spatial displacement characteristics of the accumulated temperature and precipitation contour.

### 2.4. Time Series Analysis of Spatial Position of Contour Lines

To analyze the trend, periodicity and abruptness of the space displacements of the 500 mm precipitation contour and the accumulated temperature contour, their motivations were decomposed into two directions: the meridional direction and zonal direction. Among them, the trend was explored by using the Kendall rank correlation test [27]. The period was analyzed with the maximum entropy spectrum analysis [28]. The mutagenicity was double judged by the Mann-Kendall mutation test [29,30] and the Yamamoto method [31].

### 2.5. Division of Hydrothermal Conditions Suitability for Vegetations

According to the two key elements of precipitation and accumulated temperature, which can reflect regional hydrothermal conditions, and they were sensitive to vegetation growth, so the zoning map of comprehensive suitability of precipitation and temperature can be obtained by using the reclassify and superposition calculation on the ArcGIS platform. In this reclassify method, the precipitation and accumulated temperature were divided into Grade 1 to 10 with increasing order, the 10th grade indicated the area with the highest precipitation or the highest accumulated temperature. The higher the grade, the higher the suitability. The precipitation and accumulated temperature levels were superimposed one by one on the grid unit, and the appropriate level of hydrothermal conditions in the study area can be obtained.

## 3. Results

### 3.1. Characteristics of Annual Precipitation and the 500 mm Precipitation

#### 3.1.1. Spatial Characteristics of Annual Precipitation

**Figure 3:**
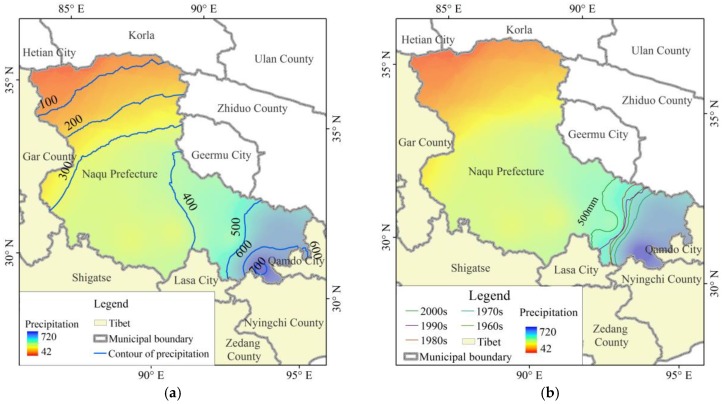
The spatial distribution of mean annual precipitation in Naqu Prefecture (**a**) and the evolution characteristics of spatial displacement of the 500 mm precipitation contours on inter-decadal time-scale (**b**).

From 1961 to 2011, the mean annual precipitation in China was 589 mm, and 342 mm in Tibet, while it was 330 mm in Naqu Prefecture, i.e., only 56% of the national average level. The mean annual precipitation of Naqu Prefecture decreased successively from the northwest area (the highest value was 720 mm per year) to the southeast area (the lowest value was 42 mm per year). The areas with mean annual precipitation of more than 500 mm were located at the southeast of Naqu Prefecture, accounting for about 12.4% of the prefecture area. On an inter-decadal time-scale, the 500 mm precipitation contour presented a slight shift to the southeast in the 1970s, and then shifted to the northwest after the 1980s (Figure 3).

**Figure 4:**
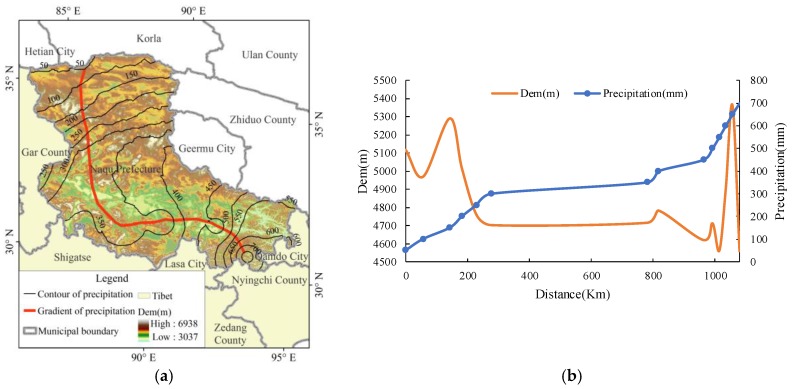
(**a**) shows the longitudinal profile of the gradient direction of the multi-year average precipitation contour. (**b**) shows the distribution of elevation and annual precipitation on the profile.

**Figure 5:**
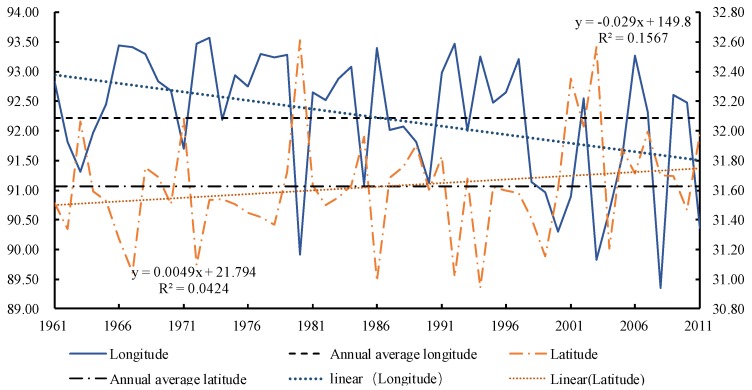
The annual movement law of the center point of the 500 mm precipitation contours.

In our study, the longitudinal profile of the gradient direction of the multi-year average precipitation contour was taken as the reference surface (Figure 4a) to analyze the distribution of elevation and annual precipitation on the profile (Figure 4b). The results showed that, along this longitudinal profile, the areas with the highest and lowest annual precipitation were located at the high-altitude areas in the region. The areas with annual average precipitation of 250 to 400 mm and elevation of 4500 to 4800 m covered the largest area. From Figure 5, it can be learned that the longitudinal profile of this part accounted for about 1/2 of the length of the entire longitudinal profile.

#### 3.1.2. Trend Analysis of the 500 mm Precipitation

Figure 5 plots the annual movement law of the center point of the 500 mm precipitation contour from 1961 to 2011. The variance range of the longitude was about 1.67°, and the 500 mm precipitation contours showed a westward shifting trend, with a rate of −0.29°/10 years. The variance range of the latitude was about 4.23°, and the 500 mm precipitation contour showed a northward shifting trend with a rate of 0.05°/10-years. The Kendall rank correlation coefficients of longitude and latitude spatial displacement were −0.1498 and 0.1624, respectively, and they were both non-significant.

#### 3.1.3. Periodicity Analysis of the 500 mm Precipitation

**Figure 6:**
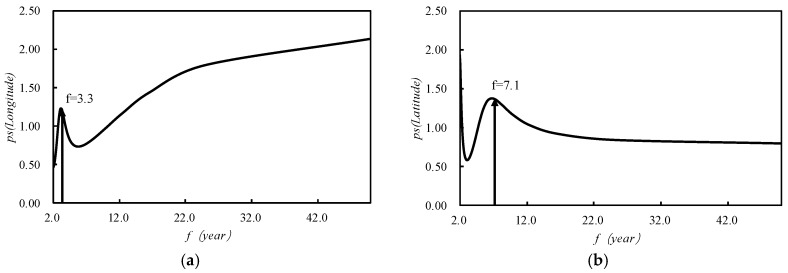
The spectrum graph of the spatial displacement of the 500 mm precipitation contour got by the maximum entropy spectrum analysis method (MESA), (**a**) is the spectrum graph of longitude direction and (**b**) is the spectrum graph of latitude direction.

Figure 6 plotted the spectrum graph of the spatial displacement of the 500 mm precipitation contour obtained by the maximum entropy spectrum analysis method. The corresponding frequency of the meridional maximum power point was 1.23, and the corresponding period length was about 3.3 years, that is to say, the meridional movement of the center point of the 500 mm precipitation contour had a 3.3-year cycle. The corresponding frequency of the zonal maximum power point was 1.37, and the corresponding period length was about 7.1 years, that is, the zonal movement of the center point of the 500 mm precipitation contour had a 7.1-year cycle.

#### 3.1.4. Catastrophe Analysis of the 500 mm Precipitation

**Figure 7:**
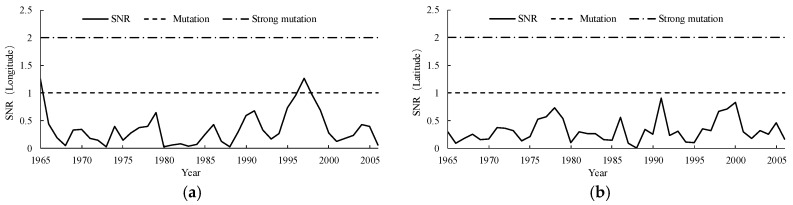
Catastrophe analysis of the center point of the 500 mm precipitation contour using the Yamamoto method, figure (**a**) shows the result of longitude direction and figure (**b**) shows the result of latitude direction.

**Figure 8:**
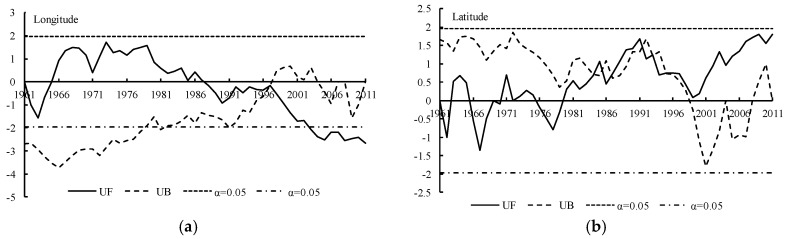
Catastrophe analysis of the center point of the 500 mm precipitation contour using the Mann-Kendall method. (**a**) The result of longitude direction and (**b**) the latitude direction result.

The Yamamoto method (Figure 7) and the Manner-Kendall method (Figure 8) were used to test the spatial displacement of the center point of the 500 mm precipitation contour. According to the Yamamoto method, no strong catastrophe point was found in the center point of the 500 mm precipitation contour series, neither in the meridional direction nor in the zonal direction. Combined with the test result of the Manner-Kendall method, it was concluded that thebcatastrophe point of the longitude of the center point of the 500 mm precipitation contour in Naqu Prefecture was around 1996, while there was no catastrophe point in the zonal direction.

### 3.2. Characteristics of Annual Accumulated Temperature and the 2000 °C Accumulated Temperature

#### 3.2.1. Spatial Characteristics of Annual Accumulated Temperature

**Figure 9:**
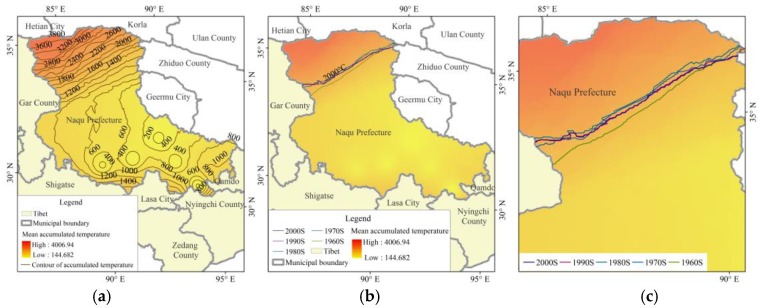
Spatial characteristics of annual average accumulated temperature (**a**) and spatial displacement of 2000 °C accumulated temperature contour (**b**), figure (**c**) is the detail draw of the 2000 °C accumulated temperature contours in different decades.

From 1961 to 2011, the mean annual accumulated nationwide temperature was 4753 °C, and the Tibet Autonomous Region one was 1693 °C, which was 35.6% of the national level, while in Naqu Prefecture, the value was only 1240 °C, which was lower than the average level of the Tibet Autonomous Region, and about 26% of the national average. The mean annual accumulated temperature of Naqu Prefecture successively decreased from the northwest area to the southeast area with a maximum value of 4007 °C and a minimum of 145 °C. The areas with mean annual accumulated temperature more than 2000 °C were located at the northwest Naqu Prefecture, accounting for about 1/5 of Naqu Prefecture. On the interdecadal scale, it appeared to shift to the northwest in the 1970s and 1980s, and to the southeast in the 1990s. After the 2000s, it continued to move to southward, but the change was small, basically coincident with the 1990s (Figure 9).

**Figure 10:**
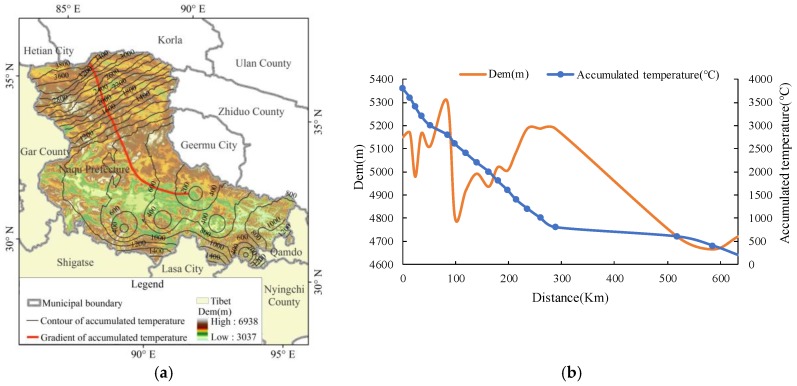
The longitudinal profile of the gradient direction of the multi-year average accumulated temperature contour (**a**) and the distribution of elevation and annual accumulated temperature on the profile (**b**).

**Figure 11:**
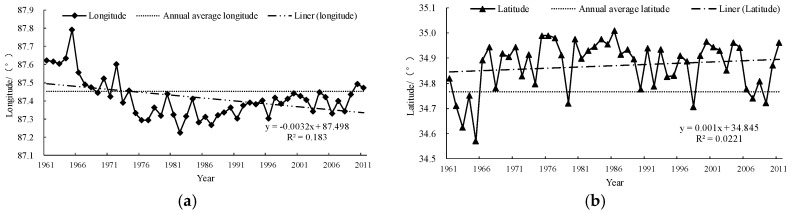
The annual movement law of the longitude (**a**) and latitude (**b**) of the center point of the 2000 °C accumulated temperature contour

Similar to the analysis method of multi-year average annual precipitation, the longitudinal profile of the gradient direction of the multi-year average accumulated temperature contour was taken as the reference surface (Figure 10a) to analyze the distribution of elevation and annual accumulated temperature on the profile (Figure 10b). Unlike the precipitation, the areas with high accumulated temperature were distributed in the relatively high or even the highest altitude areas, while the areas with the lowest accumulated temperature distributed in the lowest altitude area. The areas with the annual average accumulated temperature from 200 °C to 800 °C and the elevation from 4700 m to 5100 m covered the largest area. The longitudinal profile of this part shown in Figure 11b accounted for about 1/2 of the length of the entire longitudinal profile.

#### 3.2.2. Trend Analysis of the 2000 °C Accumulated Temperature

Figure 11 shows the annual movement law of the center point of the 2000 °C accumulated temperature contour. The variance range of the longitude was about 0.57°, and the 2000 °C accumulated temperature contour showed a westward shifting trend with a rate of −0.032°/10 years. The variance range of the latitude was about 0.44°, and the 2000 °C accumulated temperature contours showed a northward shifting trend with a rate of 0.01°/10 years. The Kendall rank correlation coefficients of longitude and latitude spatial displacement were −0.2208 and 0.19354, respectively, which were both not significant.

#### 3.2.3. Periodicity Analysis of the 2000 °C Accumulated Temperature

**Figure 12:**
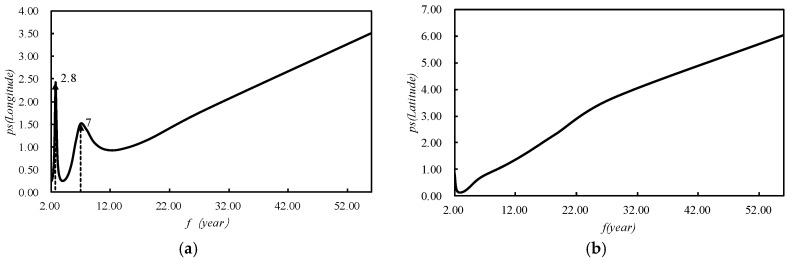
The spectrum graph of the spatial displacement of the 2000 °C accumulated temperature contour got by the maximum entropy spectrum analysis method (MESA). Figure (**a**) shows the spectrum graph of longitude direction, and figure (**b**) shows the spectrum graph of latitude direction.

From Figure 12, it can be inferred that the corresponding frequency of the meridional maximum power point was 2.42, and the corresponding period length was about 2.8 years, and corresponding frequency of the secondary meridional maximum power point was 1.51 with a corresponding period length of 7 years. That was to say, the meridional movement of the center point of the 2000 °C accumulated temperature contour had a 2.8-year period and a 7-year sub-period. The zonal movement of the center point of the 2000 °C accumulated temperature contour had no significant period.

#### 3.2.4. Catastrophe Analysis of the 2000 °C Accumulated Temperature

**Figure 13:**
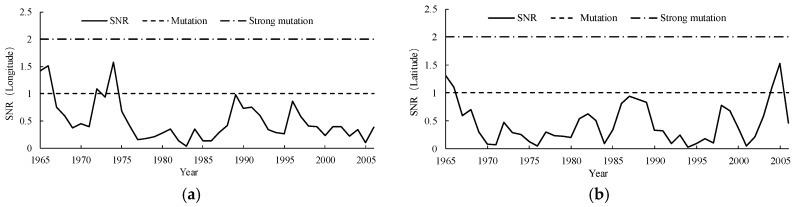
Catastrophe analysis of the center point of the 2000 °C accumulated temperature contour using the Yamamoto method. Figure (**a**) shows the result of longitude direction and figure (**b**) shows the result of latitude direction.

**Figure 14:**
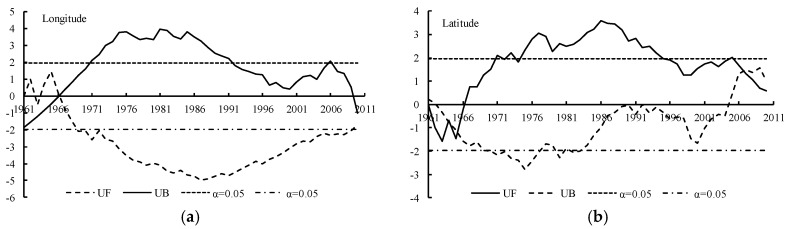
Catastrophe analysis of the center point of the 2000 °C accumulated temperature contour using the Mann-Kendall method. Figure (**a**) shows the result of longitude direction and figure (**b**) shows the result of latitude direction.

The Yamamoto method (Figure 13) and the Manner-Kendall method (Figure 14) were used to test the spatial displacement of the center point of the 2000 °C accumulated temperature contour. According to the Yamamoto method, no strong catastrophe point was found in the center point of the 2000 °C accumulated temperature contour series, neither in the meridional direction nor in the zonal direction. Combined with the test results of Manner-Kendall method, it was concluded that catastrophe point of the longitude of the center point of the 2000 °C accumulated temperature contour in Naqu Prefecture was from 1966 to 1967, while it was from 2005 to 2006 in the zonal direction.

### 3.3. Analysis of Growth Conditions of Vegetations in Naqu Prefecture

Table 1 lists the annual average precipitation and accumulated temperature of each vegetation zone in Naqu Prefecture, as well as the difference of precipitation and temperature conditions with the same vegetation zone in the rest of China. The annual precipitation and accumulated temperature values corresponding to “National” in Table 1 represent the average annual precipitation and accumulated temperature in the same vegetation zone of China, and the “difference” represents the difference of temperature or precipitation minus the national value between Naqu Prefecture and other places of China in the same vegetation zone. It can be seen from Table 1 that when in the same vegetation zone, the annual precipitation in Naqu Prefecture was generally higher than the national average, and the difference range was about 17 mm to 129 mm; while the accumulated temperature level was generally lower than the national average, the difference range was about −35 °C to −1338 °C. It was speculated from the above results that the same vegetation may require more precipitation and less accumulated temperature when grown in alpine regions, when the isothermal line of accumulated temperature and precipitation was spatially displaced, there may be a spatial redistribution of vegetation zones in Naqu Prefecture. 

### 3.4. Response Analysis of Ecosystem in Naqu Prefecture

**Figure 15:**
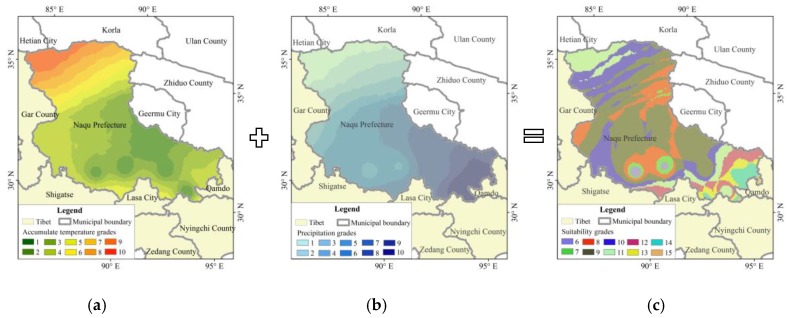
Division of hydrothermal conditions suitability of vegetations. Figure (**a**) shows the accumulated temperature grade, figure (**b**) shows the precipitation grade. Figure (**c**) shows the hydrothermal condition grade, its raster value was the sum of the raster value of figure (**a**,**b**).

According to the zoning map of comprehensive suitability of precipitation and temperature obtained by the method mentioned in Section 2.5, we can find that the suitability of vegetation growth hydrothermal conditions in Naqu Prefecture deteriorated gradually from the southeast to the northwest (Figure 15). Considering the displacement of the precipitation and accumulated temperature contours analyzed in the former sections, the center of the precipitation and accumulated temperature contours all shifted toward northwest, it is concluded that, areas with suitable hydrothermal conditions for vegetation growth in Naqu Prefecture will tend to shift toward the northwest in the future.

## 4. Discussion

### 4.1. Can Trees Be Planted in Alpine Areas Like Naqu Prefecture?

From Figure 3, Figure 4, Figure 9 and Figure 10 we find that most areas in Naqu Prefecture have an annual average precipitation of 250 mm to 400 mm, an annual accumulated temperature of 200 °C to 800 °C, both out of the threshold of the suitable hydrothermal conditions. These findings support most opinions of the related experts on “planting trees in Naqu Prefecture” [18], it may also imply that is is ossible to plant trees, especially shrubs and forests, in most alpine areas like the Tibetan Plateau.

As revealed in Section 3.1 and Section 3.2, the 500 mm precipitation and the 2000 °C accumulated temperature contours all shifted in both the longitude and latitude directions. This may be affected by the variation of South China Sea and the Indian Ocean water vapor streams [32,33]. Our results are in line with previous studies about the precipitation and temperature variation characteristics [34,35]. We also analyzed the periodicity and catastrophe of the center point of the 500 mm precipitation and 2000 °C accumulated temperature contours, and the results showed that they both have a significant moving period and catastrophe point in the longitude and latitude directions, thu indicating that in the future, under climate change conditions, the hydrothermal conditions in Naqu Prefecture may change resoundly, and there might be some areas that become suitable for trees and forest growth. This conclusion is also applicable for other regions with a similar environment on Earth.

### 4.2. Methods and Thresholds Aiming at Specific Areas Are Needed

As is shown in Table 1, for the growth of the existing vegetation in Naqu Prefecture, the same kind of vegetation may need more precipitation and lower accumulated temperature compared with the national average level. This phenomenon is probably affected by the specially high elevation, low air pressure, long-wave radiation, short-wave radiation, etc. in Tibet. Temperature and precipitation also affect the vegetation phenology and the start date of the growth season in different areas [36], as the threshold of accumulated temperature and precipitation varies in different environments and areas [37,38,39,40]. Hence, we point out that while we can’t determine whether the hydrothermal conditions are suitable for vegetation growth in alpine areas and high-altitude areas by conventional analysis, we should explore methods and thresholds aiming at these specific areas. For example, when considering the impact of precipitaion, the ratio of rain and snow, the distribution and variation of permafrost should also be taken into account, as they can also affect the distribution of swamps and grasslands [41,42].

### 4.3. Climate Change Adaptation of Ecosystem

According to the zoning map of comprehensive suitability of precipitation and temperature, the suitability of vegetation growth hydrothermal conditions in Naqu Prefecture deteriorated gradually from the southeast to the northwest. There was scant evidence for the interactive effect of warming and precipitation change on ecosystem respiration [43]. Previous studies had shown that precipitation and temperature can change the NDVI [44], and they can also affect the growth process of plants, such as seed germination [45]. Zhang et al. [46] confirmed the large spatial variability of trends in extreme precipitation indices in Tibet, and this might be affected by the surface coverage [47], which meaning that the climate change and ecosystem influence each other. Considering the displacement of the precipitation and accumulated temperature contours analyzed in the former sections, the center of the precipitation and accumulated temperature contours all shifted toward northwest, it is concluded that, areas with suitable hydrothermal conditions for vegetation growth in Naqu Prefecture will tend to shift toward the northwest in the future. This finding can provide guidelines for plant ecological system protection and climate change adaptation.

## 5. Conclusions

To reveal the changes of annual precipitation and accumulated temperature (of daily temperatures higher than 10 °C), we analyzed the spatial characteristics of the 500 mm precipitation and the 2000 °C accumulated temperature, and also the response of the ecosystem to these factors from the perspectives of trend, periodicity and abruptness was systematically explored. The results showed that:

1. On the interdecadal scale, the 500 mm annual precipitation contour in Naqu Prefecture showed a slight shift to the southeast in the 1970s, then shifted to the northwest in the 1980s. The 2000 °C accumulated temperature contour showed an offset to the northwest in the 1970s and 1980s, and moved back to the southeast in the 1990s, then continued to move southward after the 2000s.
2. The 500 mm annual precipitation contour presented a westward shifting trend and a southward shifting trend in longitude and latitude, respectively. The coordinate of the center point of the contour had a 3.3-year period and a 7.1-year period in longitude and latitude, respectively. The longitude of the center point had a catastrophe point around 1996, while there was no catastrophe point in the zonal direction.
3. The 2000 °C accumulated temperature contour showed no significant westward and northward shifting trend in the meridional and zonal directions. The center point of the contour had a 2.8-year period and a 7-year sub-period in the meridional direction, while there was no significant period in the zonal direction. The longitude of the center point had a mutation point between 1966 and 1967, and the latitude had a catastrophe point between 2005 and 2006.
4. When in the same vegetation zone, the annual precipitation in Naqu Prefecture was generally 17 mm to 129 mm higher than the national average, while the accumulated temperature level is generally 35 °C to 1338 °C lower than the national average. It was speculated that the same vegetation may require more precipitation and less accumulated temperature when grown in alpine regions. When the accumulated temperature and precipitation are spatially displaced, there may be a spatial redistribution of vegetation zones in Naqu Prefecture.
5. At present, the hydrothermal conditions suitability of vegetation growth in Naqu Prefecture gradually got worse from southeast to northwest. In the future, affected by the spatial displacement of precipitation and accumulated temperature, the areas with suitable hydrothermal conditions suitable for vegetation growth show a northwestward shifting tendency.

This research should help to deliver some recommendations for plants’ ecological system protection in Naqu Prefecture, and also provide some guidelines for climate change adaptation. However, this paper merely analyzed precipitation and accumulated temperature, and the suitable conditions for vegetation growth could also be affected by terrain factors [48,49], soil moisture [50,51], sunshine [52], wind [53,54], human activities [55,56,57], etc., so further research should also include analyses from the perspective of these aspects.

## Figures and Tables

**Table 1:** Precipitation and temperature conditions of different vegetation zones of national average level and Naqu Prefecture.

Vegetation Zones	Annual pre. (mm)			Annual Accumulated Temp. (°C)		
	Naqu	National	Diff.	Naqu	National	Diff.
Alpine grassland zone	304	266	39	1126	1218	−91
Alpine shrub meadow zone	585	566	19	849	804	45
Alpine meadow zone	478	420	57	505	541	−35
Cold-temperate coniferous forest zone	651	616	35	767	2105	−1338
Alpine desert zone	76	60	17	3389	3635	−246
Warm grassland zone	515	386	129	1034	1437	−403

**Note:** “pre.” represents “precipitation”; “temp.” represents “temperature”; “Naqu” represents “Naqu Prefecture”; “Diff.” represents “difference”.
